# The Application of Angiotensin Receptor Neprilysin Inhibitor in Cardiovascular Diseases: A Bibliometric Review From 2000 to 2022

**DOI:** 10.3389/fcvm.2022.899235

**Published:** 2022-05-04

**Authors:** Xia Xu, Yumeng Li, Shuqing Shi, Jiayu Lv, Yajiao Wang, Haoran Zheng, Xinxin Mao, Huaqin Wu, Bingxuan Zhang, Qingqiao Song

**Affiliations:** ^1^Department of General Internal Medicine, Guang’anmen Hospital, China Academy of Chinese Medical Sciences, Beijing, China; ^2^College of Traditional Chinese Medicine, China Academy of Chinese Medical Sciences, Beijing, China; ^3^College of Traditional Chinese Medicine, Beijing University of Chinese Medicine, Beijing, China; ^4^Department of Cardiology, Guang’anmen Hospital, China Academy of Chinese Medical Sciences, Beijing, China

**Keywords:** angiotensin receptor neprilysin inhibitor, cardiovascular disorders, CiteSpace, research hotspots, mapping knowledge domains

## Abstract

Cardiovascular disease (CVD) has become a huge challenge for the global public health system due to its high morbidity, mortality and severe economic burden. In recent years, angiotensin receptor neprilysin inhibitor (ARNI), a new class of drugs, has shown good therapeutic effects on CVD patients in several clinical studies, reducing the morbidity and mortality of CVD patients. In this study, we retrieved publications on ARNI research in the cardiovascular field from the Web of Science core collection and analyzed the annual output, spatial and temporal distribution, institutions and authors, core journals, keywords and co-cited literature based on CiteSpace. As a result, 604 publications were retrieved, and the number of annual publications generally increased year by year, with the largest number of articles. The analysis of the co-occurrence of output countries and authors showed that a few developed countries such as the United States, Canada, and United Kingdom are the most active in this field, forming academic groups represented by John Joseph Valentine McMurray and Scott D. Solomon, and New England Journal of Medicine, Cirulation, and Journal of the American College of Cardiology are the most popular journals in the field, with research hotspots focused on ARNI in the treatment of total ejection fraction heart failure, hypertension and its target organ damage, with the potential for future benefit throughout the cardiovascular event chain as research progresses. This study reveals the prospective application of ARNI in the cardiovascular field and the research hotspots, providing broader and deeper guidance for its use in the clinic, which is beneficial to improve the treatment and prognosis of CVD patients.

## Introduction

Cardiovascular disease (CVD) refers to a group of diseases involving the heart or blood vessels, including heart failure, hypertensive heart disease, rheumatic heart disease, stroke, and many other vascular and cardiac problems (1). In 2017, it was reported that there were approximately 485.6 million people with CVD worldwide, of which 17.8 million died from CVD, an increase of 21.1 and 28.5% (2), respectively, compared to a decade earlier. CVD has become the most common cause of death worldwide and one of the most serious health problems worldwide, accounting for 31% of all deaths worldwide (3). In addition to deaths, CVDs represent a significant economic burden, accounting for a large portion of global healthcare expenditures and lost productivity. In a recent report, the American Heart Association estimated that the healthcare costs and lost productivity of CVD are expected to grow from $555 billion in 2015 to $1.1 trillion in 2035, and with the cost of home, informal or unpaid care provided to CVD patients, the total cost of CVD will reach $1.2 trillion (4). Therefore, prevention and treatment of CVD onset, progression, and recurrence is currently a huge challenge for global public health systems.

At present, the commonly used therapeutic drugs for CVD mainly include angiotensin-converting enzyme inhibitors, angiotensin receptor antagonists, β-receptor antagonists, vasodilators, diuretics, a-receptor antagonists, positive inotropic drugs, lipid-modulating drugs, antiarrhythmics, calcium channel blockers, etc. Although they have improved the quality of life and survival rate of CVD patients to a certain extent, the action of the above drugs is relatively single, and the combination of several drugs is often required to achieve good results in the treatment of some refractory CVD. Angiotensin receptor neprilysin inhibitor (ARNI) is a dual neuroendocrine regulator with simultaneous inhibition of angiotensin receptor and enkephalinase, which is effective in treating myocardial remodeling by dilating blood vessels, promoting sodium and urine excretion, and inhibiting myocardial remodeling (5). It has shown great potential in the treatment of hypertension (6, 7), arrhythmia (8, 9), myocardial infarction (10, 11), diabetes mellitus (12, 13), and diabetic nephropathy (14, 15). Previous studies on ARNI have mostly explored one aspect of its role or mechanism, and most of the overviews of its role have been in the form of textual discussions. This review will provide the first visual analysis of ARNI research in the cardiovascular system based on CiteSpace software to analyze the current status of research in this field, research hotspots and emerging research directions over the past 20 years in a more visual way, highlighting milestone research results to help understand the development trend of this new drug and promote its widespread use in CVD patients.

## Materials and Methods

### Data Source and Search Strategy

The main databases for bibliometric analysis are Web of Science, PubMed, Cochrane Library, etc. This study chose Web of Science because the change database is a large comprehensive, multidisciplinary, high-impact academic journal, and the study confirmed that applying Web of Science database for CiteSpace visual analysis can provide better knowledge mapping effect (16, 17). The proposed search formula is: TS = (Angiotensin receptor neprilysin inhibitor OR ARNI OR Sacubitril Valsartan Sodium Tablets), refining the category: Cardiovascular System. Time span: 2000–2022; Language: English; 3 book chapters, 2 corrections, and 1 news item were excluded, and 604 relevant documents were obtained from the search. All the above operations were completed within 1 day (February 7, 2022). All records including titles, authors, abstracts, keywords, cited references, etc., were exported in plain text format and named in the format of “download_^***^.txt” as analysis data of CiteSpace.

### Scientometric Analysis Methods

CiteSpace is a Java-based application developed by Prof. Chaomei Chen at Drexel University to help visualize and analyze knowledge domains and emerging trends, showing the structure and distribution of scientific knowledge (18, 19). Some studies have shown that CiteSpace focuses on finding key points in the development of a field or domain, especially key turning points. Due to its rich functionality it has become an effective method for analyzing big data today (20, 21). After importing the data into CiteSpace, data cleaning was first performed, and then a series of network structure and temporal analyses were performed based on the data, mainly including country, institution, author, journal, keywords, and co-cited literature. The results are displayed in the form of visual graphs, where nodes represent research items, and the larger the node, the more frequently the item appears or is cited; links between nodes describe the co-occurrence or co-citation between these nodes, and the thickness of the line represents the strength of the link, and the shade or hue of the node and link color indicates the temporal order of the item’s appearance (22). Centrality is an indicator used to measure the importance of an element. If the centrality is greater than 0.1, the element is considered relatively important and is represented by a purple ring (23). Cluster analysis is also an important analysis in CiteSpace, where modularity *Q* and mean contour are two important evaluation metrics in cluster analysis, with Q > 0.3 indicating a sufficiently significant cluster structure and mean contour >0.5 indicating convincing clustering results (24). The basic parameters of CiteSpace software are set: Time Slicing: 2000–2022, Years Per Slice: 1 year; Top N per slice = 50; Pruning: Pathfinder, Pruning sliced networks, Pruning the merged networks.

## Results

### Annual Quantitative Distribution of Publications

**FIGURE 1:**
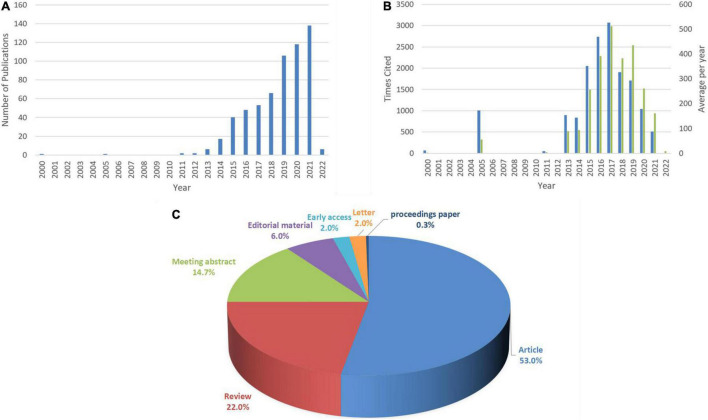
Annual number, Times Cited frequency, average per year citation frequency and document type of ARNI publications in cardiovascular system research. **(A)** Annual distribution of the number. **(B)** Blue represents Times Cited and green for average per year frequency of citations. **(C)** Type of documents.

By web of science citation analysis, the research of ARNI in the cardiovascular system showed a steady upward trend in the number of published articles per year between 2000 and 2021 (Figure 1A), which has been in a low heat period from 2000 to 2014, with fewer related studies reported and less than 30 total publications. The number of published papers increased steadily from 2015 to 2018. 40 papers were published in 2015 alone, more than the total of the previous 15 years, with an average annual growth of 12 papers and an average annual growth rate of 37.5%, indicating that the research heat and importance of this field has been noticed. The number of articles published in this field has shown an obvious upward trend in the past 3 years, with a total of more than 100 papers published per year, and has been at a high level of heat, with an average annual growth of 24 articles and an average annual growth rate of 29.6%, indicating that the research in this field has entered a period of rapid development and is being paid more and more attention. To date, these articles have been cited 15,887 times, with an average of 24.82 citations per article. Early in the study, the average annual citation frequency of articles was low due to less research on the application of ARNI in the cardiovascular system, and since 2015 the average annual citation frequency of published articles has rapidly increased and stabilized at around 300 per year, further demonstrating that research on ARNI in the cardiovascular system has entered a more mature stage (Figure 1B). The reason for this may be due to the increase in the number of patients with cardiovascular disease worldwide on the one hand and suggests a better understanding of the drug on the other. Among all published articles, ARTICLES had the highest number of 53.0%, followed by REVIEWS with 22.0% (Figure 1C). Clinical trials are one of the most important methods of medical research (25), such as randomized controlled trials and cross-sectional studies, which can assess the efficacy and safety of ARNI in order to provide useful information and guidance for the long term.

### Leading Countries

**FIGURE 2:**
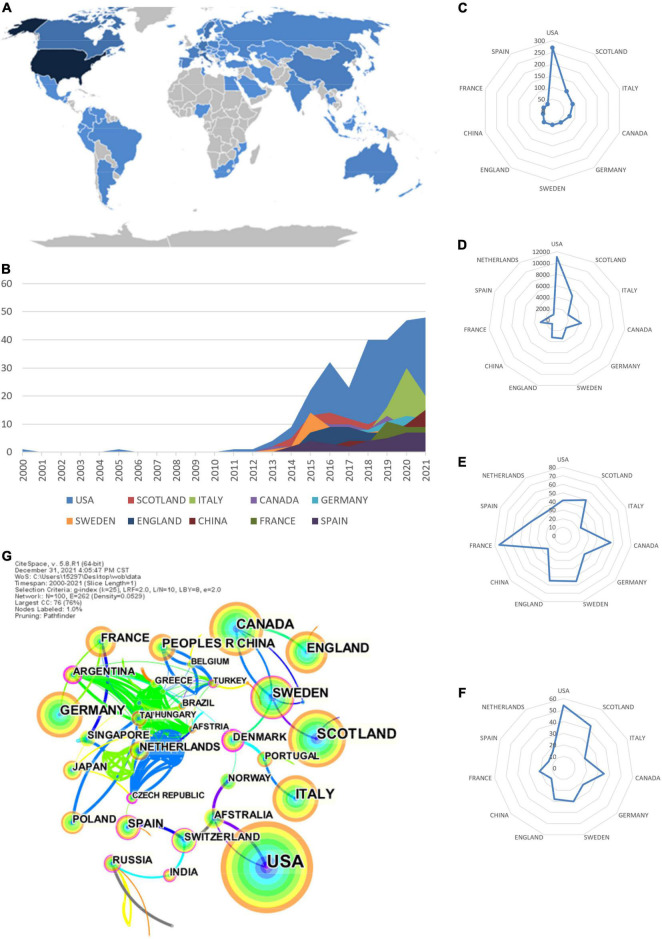
Leading countries for ARNI application research in the cardiovascular system. **(A)** Geographical distribution of global output; **(B)** Annual output trend of the top 10 productive countries; **(C)** Radar map of the top 11 productive countries; **(D)** Radar map of TGCS of the top 11 productive countries; **(E)** Radar map of average cited of the top 11 productive countries; **(F)** Radar map of h-index for the top 11 producing countries; **(G)** CiteSpace visualization of the countries participating in the ARNI in Cardiovascular Systems Study. Notes: The size of node reflects the co-occurrence frequencies, and the links indicate the co-occurrence relationships. The color of node and line represents different years, and node with purple round means high betweenness centrality (>0.1).

A total of 74 countries published articles related to ARNI research in the cardiovascular system from 2000 to 2021 (Figure 2A), and it can be seen from Figure 2 and Table 1 that the top 11 countries in terms of publications are United States (269), United Kingdom (102), Italy (91), Canada (77), and Germany (62) (Figures 2B,C), with the time of publication concentrated after 2012. The most cited country for published articles was the United States (10,913 citations), followed by United Kingdom (4,953 citations) and Canada (4,265 citations) (Figure 2D). In addition, France publications had the highest average number of citations (72.33 citations on average), followed by Canada (55.39 citations on average) and Sweden (54.87 citations on average) (Figure 2E). h-index is a new method for evaluating academic achievement, and a higher h-index indicates a more influential paper, with United States (54), United Kingdom (43) and Canada (35) were the three countries with the highest h-index (Figure 2F). The national co-occurrence network visualized in Figure 2G contains 100 nodes and 262 links, which are often considered as important turning points that may lead to transformative discoveries and act as bridges in terms of their high intermediary centrality (≥0.10) (22, 26), as seen in the figure, Denmark (0.32) ranks first, followed by Argentina (0.31) and Czechia (0.20), implying that they play an important role in expanding knowledge to other countries. Combining these three paper evaluation indicators shows that United States, United Kingdom, and Canada are the three most influential countries in this research area. However, it is worth noting that there is less cooperation between these countries and other countries, and the cooperation between countries should be strengthened in the future to drive the development of this field.

**TABLE 1:** Top 11 producing countries studied by ARNI in the cardiovascular system.

Rank	Country	Publication	TGCS	Average citation	h-index	Centrality
1	United States	269	10,913	41.2	54	0.00
2	United Kingdom	102	4,953	49.43	43	0.03
3	Italy	91	2,166	22.8	20	0.00
4	Canada	77	4,265	55.39	35	0.09
5	Germany	62	2,114	33.03	22	0.00
6	Sweden	61	3,430	54.87	30	0.19
7	England	60	3,217	54.53	28	0.03
8	China	42	1,071	23.28	14	0.00
9	France	39	2,922	72.33	21	0.03
10	Spain	34	1,364	40.12	14	0.18
11	Netherlands	31	1,117	33.85	17	0.00

### Active Institutes

**FIGURE 3:**
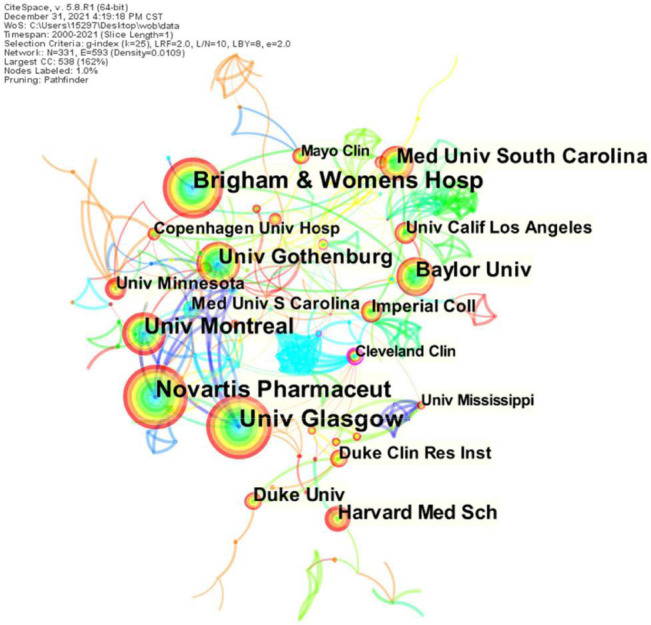
CiteSpace visualization of institutions participating in ARNI’s research in the cardiovascular system. The circular nodes represent institutions; the links between nodes represent interactions between institutions.

A total of 332 institutions published articles on the use of ARNI in the cardiovascular system (Figure 3). The top 10 institutions in terms of research output are shown in Figure 3 and Table 2, with University of Glasgow (*n* = 97) leading in terms of output, followed by Novartis Pharmaceuticals (*n* = 90), Brigham and Women’s Hospital (*n* = 87), University of Montreal (*n* = 58), and University of Gothenburg (*n* = 48). The top three institutions in terms of centrality were Cleveland Clinic (0.23), Associazione Nazionale Medici Cardiologi Ospedalieri (0.18), and Charles University Prague (0.17). As shown in the figure, there is closer cooperation between institutions.

**TABLE 2:** Top 10 institutions involved in ARNI cardiovascular system research in terms of volume and centrality of publications.

Rank	The name of institution	Publication	Rank	The name of institution	Centrality
1	University of Glasgow	97	1	Cleveland Clinic	0.23
2	Novartis Pharmaceut	90	2	Associazione Nazionale Medici Cardiologi Ospedalieri	0.18
3	Brigham and Women’s Hospital	87	3	Charles University Prague	0.17
4	University of Montreal	58	4	Glenfield Hospital	0.16
5	University of Gothenburg	48	5	Charité – University Medicine Berlin	0.13
6	Baylor University	44	6	Heidelberg University	0.12
7	Medical University of South Carolina	42	7	Meander Medical Center	0.11
8	Harvard Medical School	36	8	Semmelweis University	0.10
9	Duke University	30	9	National and Kapodistrian University of Athens	0.10
10	Imperial College London	21	10	Harvard Medical School	0.09

### Authors and Co-cited Authors

**FIGURE 4:**
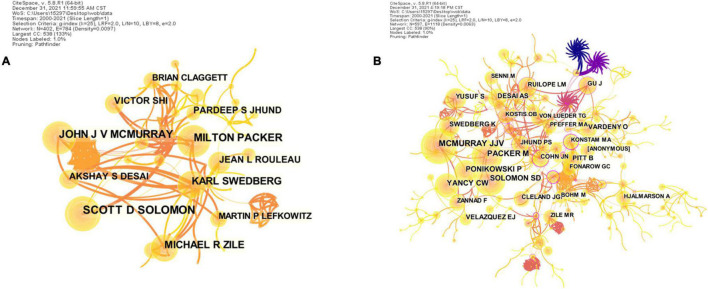
CiteSpace visualization of the authors **(A)** and co-cited authors **(B)** involved in ARNI research in the cardiovascular system. Circle node represent author of papers; link between nodes represent partnerships.

A total of 402 authors published articles on ARNI in cardiovascular system research, with the three most prolific authors being Scott D. Solomon (75 articles), Milton Packer (65 articles) and John Joseph Valentine McMurray (61 articles) (Figure 4A and Table 3). Of note is the relatively low centrality of authors (<0.03), indicating that the impact of authors on ARNI research in the cardiovascular system needs to be improved, with each node representing one author and the larger the node the more articles published. The thicker lines represent closer collaboration between authors, and it is clear from Figure 4A that there is communication and collaboration between authors in the field. Co-cited authors are those authors who are co-cited in publications, and co-citation is a key indicator of author contribution (27). The top 10 co-cited authors are shown in Figure 4B and Table 4, only nine authors have more than 100 citations, with John Joseph Valentine McMurray (403) ranked first, followed by Milton Packer (225), Clyde W. Yancy (202) and Scott D. Solomon (198). The highest centrality was Konstam M. A. (0.38), Massie B. M. (0.32), and Cohn J. N. (0.31). John Joseph Valentine McMurray, an early investigator of ARNI studies in the cardiovascular system, conducted the PARADIGM-HF study (28) in 2013, showing that LCZ696, compared with the high-dose ACEI analog enalapril, was associated with a reduction in cardiovascular mortality and the primary endpoint (the composite endpoint of cardiovascular death and heart failure hospitalization). Based on this study, in 2014 the Canadian Cardiovascular Society (CCS) (29) updated its guidelines for the management of patients with heart failure, recommending for the first time a dual angiotensin receptor and enkephalinase inhibitor (ARNI). In the United States, Prof. Scott D. Solomon has been a driving force in this field for more than 10 years, conducting studies on the therapeutic role of sacubitril valsartan in heart failure and accelerating the use and dissemination of this drug in clinical care. However, their low centrality indicates that they have less interaction with other authors and do not play an important role in expanding their knowledge to other authors.

**TABLE 3:** Top 10 authors in terms of number and centrality of articles related to ARNI in cardiovascular system research.

Rank	Authors	Publication	Centrality
1	Solomon S. D.	75	0.01
2	Packer M.	65	0.02
3	McMurray J. J. V.	61	0.02
4	Michael R. Zile	51	0.00
5	Karl Swedberg	47	0.00
6	Akshay S. Desaj	38	0.01
7	Jean L. Ronarow	37	0.01
8	Pardeep S. Jhund	33	0.00
9	Victor C. Shi	31	0.01
10	Martin Lefkowitz	29	0.00

**TABLE 4:** Top 10 co-cited authors in terms of number and centrality of articles related to ARNI in cardiovascular system research.

Rank	Co-cited authors	Count	Rank	Co-cited authors	Centrality
1	McMurray J. J. V.	403	1	Konstam M. A.	0.38
2	Packer M.	225	2	Massie B. M.	0.32
3	Yancy C. W.	202	3	Cohn J. N.	0.31
4	Solomon S. D.	198	4	Arnett D. K.	0.30
5	Ponikowski P.	183	5	Messerli F. H.	0.29
6	Desai A. S.	112	6	Borlafg B. A.	0.23
7	Yusuf S.	109	7	Backlund T.	0.20
8	Pitt B.	102	8	Rouleaf J. L.	0.19
9	Vardeny O.	101	9	Campbell D. J.	0.18
10	Swedberg K.	98	10	Cannon J. A.	0.17

### Core Journals

**FIGURE 5:**
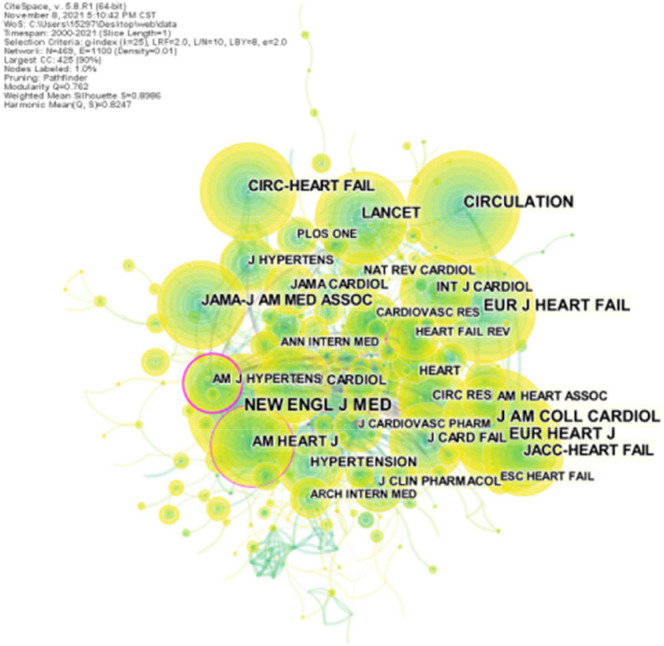
CiteSpace visualization of co-cited journals involved in ARNI research in the cardiovascular system. The circle nodes represent co-cited journals; the links between nodes represent interdisciplinary interactions in the literature.

**FIGURE 6:**
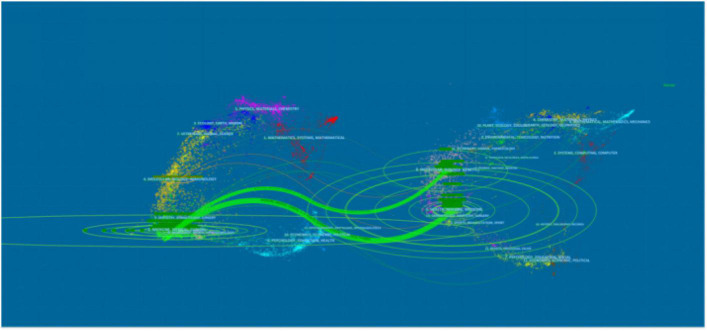
The dual-map overlay of citations of articles on the application of ARNI in the cardiovascular field (The left side were the citing journal, the right side were the cited journal, and the line path represents the citation relationship).

All articles on ARNI research in the cardiovascular field were published in a total of 102 journals. The top 5 most cited journals and the top 5 journals with the highest centrality were found through journal co-citation co-presentation shown in Table 3, New England Journal of Medicine (452 total citations) was the most prolific journal followed by Cirulation (413 total citations) and Journal of the American College of Cardiology (377 total citations). The journal with the highest centrality was American Journal of Hypertension (0.37), followed by American Heart Journal (0.12), and Journal of Cardiovascular Medicine (0.10) (Figure 5 and Table 5), all three with good centrality (≥0.01), indicating that they are the more influential journals. The double map overlay shows the two main citation paths. The published articles were concentrated in journals in the fields of medicine, medical, and clinical, while the cited articles were mostly published in journals in the fields of molecular, biology, genetics, health, nursing, and medicine (Figure 6).

**TABLE 5:** The top 5 core and the top five centrality journals on ARNI study in the cardiovascular system.

Rank	Journal	Count	Rank	Journal	Centrality
1	New England Journal of Medicine	452	1	American Journal of Hypertension	0.37
2	Cirulation	413	2	American Heart Journal	0.12
3	Journal of the American College of Cardiology	377	3	Journal of Cardiovascular Medicine	0.10
4	European Heart Journal	365	4	Chest	0.09
5	Lancet	360	5	Journal of Biological Chemistry	0.08

### Analysis of Keywords

**FIGURE 7:**
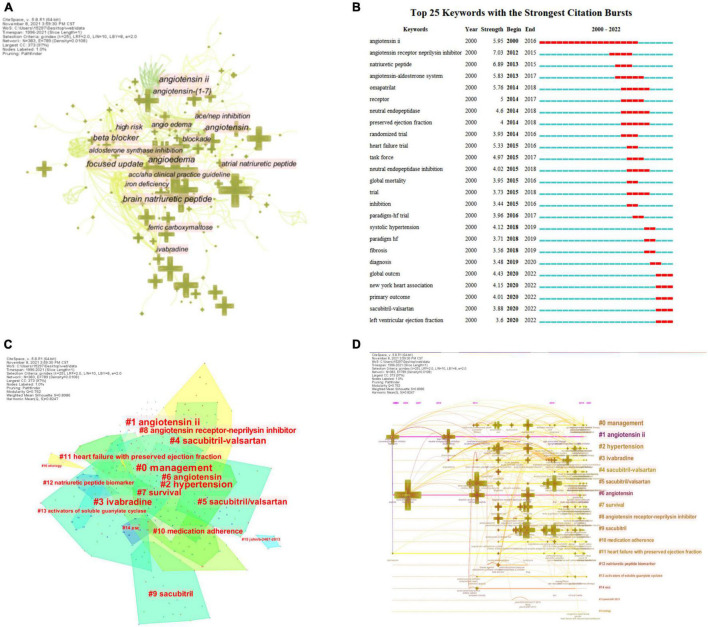
CiteSpace visualization of keywords involved in ARNI research in the cardiovascular system. **(A)** Network of the main keywords in publications on ARNI in cardiovascular system research and related research. **(B)** Time trend graph of burst keywords. **(C)** Cluster analysis of keywords. **(D)** The timeline view of Keyword clusters.

Keywords are condensed and extracted from the content of an article to reflect the topic and content of the representative article, and high-frequency keywords are often used to reflect hot issues in the research field (30). Keyword co-occurrence network is a text content-based analysis method, and we extracted 383 keywords at the time of data collection (Figure 7A), and the top 25 most cited keywords were found by keyword burst (Figure 7B); the blue line represents the time interval, and the red segment represents the burst cycle time of the keywords; from the figure, we can see that the burst keywords are mainly focused on ARNI and the treatment of related disease studies, angiotensin II, angiotensin receptor neprilysin inhibitor, randomized trial, heart failure trial, neutral endopeptidase inhibition, global mortality began to break out in the early stages. Thereafter fibrosis, systolic hypertension, and PARADIGM-HF trials began to explode; in recent years, sacubitril-valsartan, and left ventricular ejection fraction have become new keywords for the outbreak.

Clustering analysis based on log likelihood ratio (LLR) revealed a total of 17 clusters with a modularity Q of 0.762 and a mean profile value of 0.8986, showing good homogeneity (31). They contain a large number of concerns of ARNI research in the cardiovascular field, including drugs [#1 angiotensin II (32), #3 ivabradine (33), #4 sacubitril-valsartan (34), #5 sacubitril/valsartan (35), #6 angiotensin (36), #8 angiotensin receptor-neprilysin inhibitor (37), #9 sacubitril (38)], indications [#2 hypertension (39), #11 heart failure with preserved ejection fraction (40)], management [#0 management (41)], guidelines [#14 ESC (42)], etiology [#16 etiology (43)], and observables [#7 survival (44), #10 medication adherence (45), #12 natriuretic peptide biomarkers (46)] (Figures 7C,D). In recent years, due to the emergence of some important new evidence-based medicine for heart failure drugs, the 2021 ESC guidelines (47) have been updated in this regard, and the status of ARNI has been importantly elevated to become the first-line drug for heart failure treatment, which brings hope for the treatment of heart failure patients. Not only that, several clinical studies in recent years have found that ARNI, represented by sacubitril valsartan, has good therapeutic effects in lowering blood pressure as a new generation drug, and its mechanism of action mainly includes diuretic, natriuretic, vasodilator and anti-sympathetic activity (48–53). Based on this, the 2020 International Society of Hypertension (ISH) Global Hypertension Practice Guidelines first recommended it as the preferred antihypertensive treatment for heart failure patients, bringing new hope for the treatment of hypertensive patients.

### Co-cited References

**FIGURE 8:**
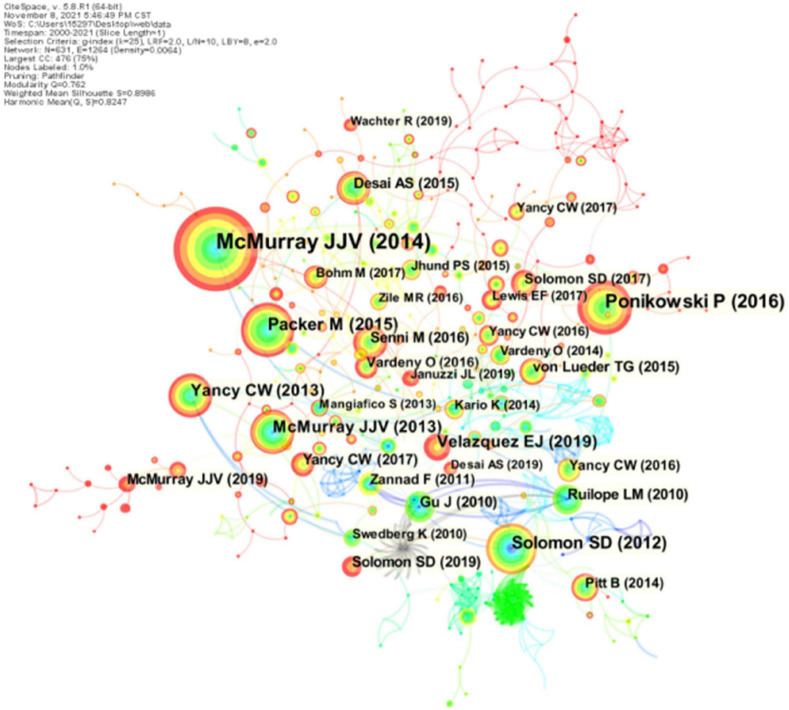
CiteSpace visualization of the co-cited reference involved in ARNI studies in the cardiovascular system. Circle node represents reference; the line between the nodes indicates the frequency of the two references being cited at the same time. The betweenness centrality of the nodes with red circle is >0.1.

Co-citation analysis was proposed by Small and later introduced to the co-citation analysis of references, the phenomenon that two or more references are cited in the same literature (54), and the analysis of literature with higher co-citation frequency and centrality can obtain the disciplinary research base, which was obtained after CiteSpace analysis for the last 21 years of ARNI and The visualization of the co-cited literature in the field of cardiovascular disease-related research for the last 21 years (*N* = 631, *E* = 1264). N denotes nodes/number and E denotes the concatenation of objects between nodes. As can be seen, *N* = 631 and *E* = 1264 in the co-cited literature mapping, indicating that there are 631 co-cited References included in the mapping, each node represents a co-cited literature, and the line between the nodes represents the cross-citation between references, indicating that there are 1,264 citations between references. As shown in Figure 8, in which the circular nodes represent references, and the larger the node, the higher the citation frequency of the reference literature. Of the top 10 co-cited articles, 8 were clinical studies and 2 were guidelines for the management of heart failure (Table 6). The clinical studies focused on the safety and efficacy of ARNI in the treatment of patients with various types of heart failure, and their pharmacokinetics and pharmacodynamics were investigated. It can be seen that the study of the therapeutic effects of ARNI in heart failure remains a major aspect of current research.

**TABLE 6:** Top 10 most cited papers.

Variable	First author	Journal	Year	TGCS
1	McMurray J. J. V.	New England Journal of Medicine	2014	358
2	Ponikowski P.	European Heart Journal	2016	153
3	Solomon S. D.	Lancet	2012	105
4	Packer M.	Circulation	2015	103
5	McMurray J. J. V.	European Journal of Heart Failure	2013	80
6	Yancy C. W.	Journal of the American College of Cardiology	2013	72
7	Velazquez E. J.	New England Journal of Medicine	2019	71
8	Solomon S. D.	New England Journal of Medicine	2019	53
9	Desai A. S.	European Heart Journal	2015	51
10	Gu J.	Journal of Clinical Pharmacology	2010	49

**FIGURE 9:**
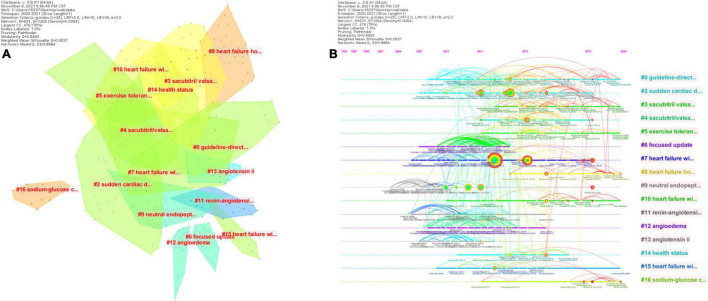
CiteSpace visualization of the cluster analysis of the co-cited literature involved in ARNI studies in the cardiovascular system. **(A)** Cluster analysis of co-cited references; **(B)** Timeline distribution of clusters.

A subsequent cluster analysis of the cited references identified 16 clusters with a modularity *Q* of 0.8492 and an average profile value of 0.9537 (Figure 9A and Table 7). Based on this, a visual timeline was executed for the clusters, combining clustering and time slicing techniques to not only reflect the distribution of topics in the field, but also to depict the trends and interconnections of the research topics over time (Figure 9B). The blue and green colors in the figure indicate literature from earlier times, while the yellow and red nodes represent more recent literature. As seen in the figure, early studies focused on the therapeutic mechanisms, indications and possible adverse effects of ARNI in cardiovascular disease (Cluster #4, Cluster #6–10). With the accumulation of strong evidence, this question has been largely answered and the focus of research has therefore shifted to the designation of therapeutic specifications for ARNI in cardiovascular disease (cluster #0) and new combination therapeutics (cluster #16), among others. Notably, in recent years SGLT2i, a novel agent, has been shown to increase urinary glucose excretion, urinary sodium excretion and osmotic diuresis, improve myocardial energy metabolism, inhibit sympathetic activity, modulate the cardiac inflammatory response, improve ventricular remodeling, and improve cardiac function, with benefits for the cardiac-renal-metabolic system (55). 2021 ESC guidelines (29) even emphasize that all HFrEF patients should receive a combination of four drugs: ACEI/ARNI, β-blocker (BB), aldosterone receptor antagonist (MRA), and SGLT2. In conclusion, the co-citation analysis of ARNI references in the cardiovascular field is a way to learn more about the evolution and hotspots of this research field and helps to identify the core topics and concerns in the field.

**TABLE 7:** The 10 co-cited reference clusters of ARNI studies in the cardiovascular system.

Cluster ID	Size	Silhouette	Mean year	Top terms
#0	61	0.903	2015	Guideline-directed medical therapy
#2	43	0.952	2015	Sudden cardiac death
#4	36	0.983	2016	Sacubitril/valsartan
#6	32	0.976	2012	Focused update/angioedema
#7	32	0.944	2013	Heart failure with preserved ejection fraction
#8	30	0.985	2019	Heart failure hospitalization
#9	29	1	2008	Neutral endopeptidase
#10	26	0.99	2017	Heart failure with reduced ejection fraction patients
#11	21	0.955	2011	Renin-angiotensin-aldosterone blockade
#16	14	1	2018	Sodium-glucose cotransporter-2 inhibitors

## Discussion

This study analyzed the knowledge hotspots and new development trends of ARNI research in the cardiovascular field using bibliometric methods, and found that the number of publications in this field showed a yearly increase and entered a more mature stage of development, but there is still an imbalance in its development between regions and countries, with only one developing country, China, among the top 10 countries in terms of the number of publications, which indicates that the research in this field in the past 20 This indicates that Chinese research in this field has made great progress over the past 20 years. However, the vast majority of research is concentrated in developed countries such as the United States, the United Kingdom, and Canada, and accordingly these countries have formed several of the most active academic groups, with Prof. Scott D. Solomon’s group at Brigham and Women’s Hospital in the United States, Prof. Milton Packer’s group at Baylor University Medical Center in Dallas, and Prof. John G. G. Gutierrez’s group at University of Glasgow in the United Kingdom, but the low centrality of these authors suggests that they do not play a significant role in expanding their knowledge to other authors, and therefore communication and collaboration between authors should be enhanced to promote the field.

The top three journals in terms of IF are New England Journal of Medicine (IF 2021 = 91.24), Lancet (IF 2021 = 79.321) and JAMA- Journal of the American Medical Association (IF 2021 = 56.272). It is suggested that ARNI research articles in the cardiovascular field are mainly published in journals with higher impact factors and they are cited more frequently. IF is a quantitative indicator representing the impact size of journals, reflecting the citation rate, academic level and paper quality, indicating that research papers in this field are of high quality and provide strong evidence for the clinical application of the drug.

According to the analysis, we got that ARNI research in the field of heart failure is the main research direction, and there are three important points in its development history, the first one is the 2014 Canadian Cardiovascular Society (CCS) updated the guidelines for the management of heart failure patients based on the results of the PARADIGM-HF study, which for the first time The second is the update of the 2016 ACC/AHA/HFSA (56) heart failure guidelines, based on the results of the PARADIGM trial, which recommended ARNI as a class I drug, the first time recommending ARNI as an alternative to ACEI therapy for patients with poor outcomes. Drug for reducing mortality in some patients with chronic heart failure (HFrEF). This guideline update not only upgraded the recommendation level of ARNI class of drugs in the cardiovascular field, but also clearly indicated the scope of application of the drug, providing clearer guidance for clinical use; the third is a major update of the 2021 ESC heart failure guideline based on the PIONEER-HF study (57) and the PARAGON-HF study (40), which strengthened the first-line recommendation of ARNI status, with sacubitril valsartan available as a starting drug in HFrEF patients who have not previously used an ACEI and, for the first time, including sacubitril valsartan as a therapeutic agent for HFmrEF, and with the PARAGON-HF study confirming that ARNI reduces the composite endpoint of heart failure hospitalization and cardiovascular death in HFpEF patients by 13%. With the approval of the HFpEF indication by the FDA, this makes ARNI the only class of drugs that can treat total ejection fraction heart failure, broadening the therapeutic scope of the drug and thus better guiding clinical practice.

As research on this class of drugs continues, the effectiveness of ARNI in the treatment of other cardiovascular diseases is becoming a topic of interest for researchers, with an important keyword cluster being hypertension. Notably, the first antihypertensive study of ARNI was published in Lancet in 2010, and its results showed that ARNI could dose-dependently lower patients’ blood pressure and was superior to valsartan (58). Several subsequent studies have found that ARNI is not only effective in controlling blood pressure, but also improves it in patients with hypertension combined with left ventricular hypertrophy, coronary artery disease, and chronic kidney disease. For example, the EVALUATE-HF study and the PROVE-HF study found that sacubitril valsartan treatment of HFrEF patients resulted in rapid improvement of cardiac remodeling indexes such as left ventricular end-systolic volume index (LVESVi), left ventricular end-diastolic volume index (LVEDVi), left atrial volume index (LAVI), and left ventricular mass index (LVMI), with 1 year of treatment consistently reversing cardiac remodeling (59, 60). Subgroup analysis of the PARADIGM-HF study showed that sacubitril valsartan significantly reduced the risk of the composite coronary endpoint (cardiovascular death, non-fatal myocardial infarction, unstable angina, other angina hospitalization, or coronary revascularization) by up to 17% compared with ACEI (46). In a study of an Asian population with hypertension combined with CKD, sacubitril valsartan reduced systolic blood pressure by 20.5 mm Hg and urinary albumin/creatinine ratio (UACR) by a significant 15.1% in patients with hypertension combined with CKD (61). In the future, it is believed that sacubitril valsartan will fill more gaps in the full management of the cardiovascular event chain, giving full benefits to cardiovascular patients.

This study is based on the high morbidity and mortality of cardiovascular disease and the serious economic burden it brings to study the value of the application of ARNI. Through this study, it is found that ARNI can effectively improve the long-term prognosis of patients with cardiovascular disease, and at the same time, the drug can act on the whole course of cardiovascular events, helping to delay the progression of the disease and reduce the occurrence of adverse cardiovascular events. The use of this drug can also improve the quality of life of such patients, reducing the costs required for hospitalization and care, and reducing the economic burden on families and society. This shows that the study of this drug in the cardiovascular system brings real economic benefits and social impact.

There are also certain shortcomings in this study. First, this study mainly uses Web of Science core database for literature search, although it enriches the search strategy as much as possible, there is still a possibility that relevant literature is not included; in addition, this study mainly uses CiteSpace for literature analysis, and the difference in the quality of the retrieved literature data will lead to a reduction in the quality and credibility of the plotted images, and combining the two methods of CiteSpace and VOSviewer can provide a more accurate analysis of the literature.

## Conclusion

The bibliometric analysis revealed a promising research landscape for ARNI in the cardiovascular field with a significant increase in related publications. The main contributors to this research area were clearly identified, and it was found that the relevant literature mainly focuses on the application of ARNI in heart failure, hypertension and other diseases, which may benefit in the future throughout the cardiovascular event chain. However, regional and national differences still exist, and there is a need to strengthen cooperation between countries. It is hoped that this study will lead to a greater understanding of the prospects of ARNI in the cardiovascular field and promote its clinical application for better treatment and prognosis of CVD patients.

## Data Availability Statement

The original contributions presented in the study are included in the article/supplementary material, further inquiries can be directed to the corresponding author/s.

## Ethics Statement

Written informed consent was not obtained from the individual(s) for the publication of any potentially identifiable images or data included in this article.

## Author Contributions

QS, XX, and SS devised the research plan and established the methodology. XX, SS, YL, JL, and YW wrote the original draft. HZ and XM were in charge of software, literature retrieval, and visualization. HW, BZ, and XX modified and polished the manuscript. All authors contributed to the article and approved the submitted version.

## Conflict of Interest

The authors declare that the research was conducted in the absence of any commercial or financial relationships that could be construed as a potential conflict of interest.

## Publisher’s Note

All claims expressed in this article are solely those of the authors and do not necessarily represent those of their affiliated organizations, or those of the publisher, the editors and the reviewers. Any product that may be evaluated in this article, or claim that may be made by its manufacturer, is not guaranteed or endorsed by the publisher.
